# Correction to Machner et al. (2018)

**DOI:** 10.1037/neu0000541

**Published:** 2019-05

In the article “The Ipsilesional Attention Bias in Right-Hemisphere Stroke Patients as Revealed by a Realistic Visual Search Task: Neuroanatomical Correlates and Functional Relevance” by Björn Machner, Inga Könemund, Janina von der Gablentz, Paul M. Bays, and Andreas Sprenger (*Neuropsychology*, Volume 32, Issue 7, October 2018 http://dx.doi.org/10.1037/neu0000493) the copyright attribution was incorrectly listed and the Creative Commons CC-BY license disclaimer was incorrectly omitted from the author note. The correct copyright is “© 2018 The Author(s)” and the omitted disclaimer is below. The online version of this article has been corrected.

“This article has been published under the terms of the Creative Commons Attribution License (http://creativecommons.org/licenses/by/3.0/), which permits unrestricted use, distribution, and reproduction in any medium, provided the original author and source are credited. Copyright for this article is retained by the author(s). Author(s) grant(s) the American Psychological Association the exclusive right to publish the article and identify itself as the original publisher.”

